# Optimization of Oligomer Stamping Technique for Normally Closed Elastomeric Valves on Glass Substrate

**DOI:** 10.3390/mi14091659

**Published:** 2023-08-25

**Authors:** Joel Dungan, Juanita Mathews, Michael Levin, Valencia Koomson

**Affiliations:** 1 Electrical Engineering Department, Tufts University, Medford, MA 02155, USA; 2 Biology Department, Tufts University, Medford, MA 02155, USAmichael.levin@tufts.edu (M.L.); 3 Wyss Institute, Harvard University, Cambridge, MA 02138, USA

**Keywords:** microfluidics, PDMS, pneumatic valve, electrical impedance, plasma bonding

## Abstract

Microscale elastomeric valves are an integral part of many lab-on-chip applications. Normally closed valves require lower actuation pressures to form tight seals, making them ideal for portable devices. However, fabrication of normally closed valves is typically more difficult because the valve structure must be selectively bonded to its substrate. In this work, an oligomer stamping technique for selective bonding of normally closed valves is optimized for bonding of PDMS devices on glass substrates. Contact angle and blister bursting testing measurements are used to quantitatively characterize the oligomer stamping process for the first time, and recommendations are made for plasma treatment conditions, microstamping technique, and valve construction. Glass–PDMS devices are ideal for lab-on-chip systems that integrate electrodes on the rigid glass substrate. Here, integrated electrodes are used to assess valve performance, demonstrating electrical isolation in excess of 8 MΩ over the biologically relevant frequency range in the closed state. Further, electrical measurement is used to demonstrate that the valve design can operate under a pulsed actuation scheme, sealing to withstand fluid pressures in excess of 200 mbar.

## 1. Introduction

Microfluidic devices have become indispensable research and diagnostic tools across a wide variety of applications. In research settings especially, the elastomer polydimethylsiloxane (PDMS) is a very popular material for microfluidic fabrication and rapid prototype development. PDMS has favorable mechanical and optical properties for many applications, and the fabrication process used to produce such devices is both low-temperature and low-cost. This “soft lithography” approach uses standard lithographic techniques to produce master molds from which many PDMS devices can be replicated by casting. A crucial quality of PDMS is the relative ease with which devices can then be bonded to a substrate, thereby sealing off microfluidic channels.

PDMS can be permanently bonded to many substrates including glass and PDMS itself. Irreversible covalent bonding occurs when sufficient energy is applied to the PDMS to activate silanol groups on the surface. Often, this energy is applied in the form of exposure to air or oxygen plasma created in the proximity of a high-frequency electrode, though surface modification through chemical treatment is also possible. Under the right conditions, these surface chains can covalently bond with other silanol groups on the substrate, releasing water. Surface activation also results in reduced hydrophobicity [1,2]. Alternate PDMS bonding methods include partial curing, cross-linker diffusion, and chemical adhesive techniques in the case of PDMS–PDMS bonding [3] and UV/ozone treatment [4] for PDMS–glass bonding. However, no bonding approach inherently enables selective bonding with microscale resolution.

Since their introduction, elastomeric valves have enabled a new generation of lab-on-chip microfluidic devices. The Quake group first presented pneumatically actuated positive-pressure microfluidic valves for PDMS devices that are normally open, demonstrating that they could be used for effective fluid control and peristaltic pumping [5]. Many other microvalve designs have since been proposed that rely on positive and negative pressure [6], including doormat [7], curtain [8,9], and plunger styles [10]. Each valve type has particular strengths and weaknesses in terms of leakage, actuation pressure, and fabrication complexity and should be selected to fit the particular application. Normally closed vacuum-actuated valves are particularly appealing for portable applications. The primary drawbacks of such valves are that some positive pressure may be required to maintain the valve seal under heavy fluid pressure, and that their fabrication technique is more complicated than their normally open counterparts as it requires selective PDMS bonding.

Previous approaches to normally closed valve fabrication have required vacuum to be applied to pull the surface in the valve area away from the substrate to prevent bonding [8]. These approaches do not scale well to lab-on-chip devices with many valves, as managing vacuum lines can be cumbersome and their weight can pull and stretch the PDMS device during bonding. Further, thermal expansion of trapped gasses in the bonding process can make vacuum levels fluctuate problematically. Proposed solutions include the use of passivation layers [11], soluble masks [10], and metal masking [9]. These approaches have shortcomings in terms of scalability and added fabrication complexity. Takayama [12] presented a compelling approach based on an oligomer stamping method for PDMS–PDMS devices. The technique requires no additional chemicals or tools: rather a PDMS stamp is applied to the plasma treated bonding surface. Oligomer chains on the inactivated PDMS stamp remove the activated surface groups on the device to be bonded, leading to highly localized regions of reduced bond strength.

Takayama’s approach allows the scalable fabrication of high-density valved devices suitable for lab-on-chip applications. Here, we further investigate this technique, characterizing the oligomer stamping process quantitatively over a large parameter space through contact angle and blister burst testing for the first time. Further, we expand the previous approach to seek treatment and stamping conditions optimal for use with a rigid glass substrate. Such substrates easily facilitate the integration of electrodes into devices, which is essential for applications such as electrophoresis, electrical impedance tomography, and impedance-flow cytometry. Additionally, glass substrates are often ideal or necessary for optical imaging. For biomicrofluidic devices, cell culture protocols are often optimized for use on rigid substrates. Integrated electrodes enable characterization of valve performance over a broad frequency range as well as leakage current monitoring. Valves fabricated using the optimized oligomer stamping technique are demonstrated to form strong seals withstanding fluid pressures greater than 200 mbar without any leakage current.

## 2. Materials and Methods

### 2.1. Fabrication Techniques

All device fabrication was performed in the Tufts Micro/Nanofab. Devices were constructed from polymethylsiloxane (PDMS, Dow Chemical Sylgard 184) at a 10:1 ratio of base to curing agent. All PDMS was degassed for approximately 30 min prior to molding and was fully cured in an oven at 60 °C for approximately 1.5 h. All PDMS surfaces intended for bonding experiments (including those without channels) were molded against silicon wafers to ensure maximum surface flatness and uniformity. Master molds were fabricated using standard lithography techniques using SU8-3050 (Microchem). All 65 µm-thickness molds were spincoated on glass substrates at 2200 rpm for 30 s, soft baked at 95 °C for greater than 15 min, and exposed to UV radiation (20 mW/cm${}^{2}$ at I-line) for 15 s (or 10 s for silicon substrates) before post-exposure baking (4 min at 95 °C) and agitated development for more than 10 min. Additionally, 100 µm stamp molds were spun at 1000 rpm for 60 s, soft baked for greater than 30 min, UV exposed for 18 s, post-exposure baked for 5 min, and developed for more than 15 min in agitated SU8 developer. To ensure easy mold release, masters were silanized by exposure to tridecafluoro-1,1,2,2-tetrahydrooctyl trichlorosilane (Gelest Inc., Morrisville, PA, USA) for one hour in a desiccator. Glass substrates were cleaned in a 3:1 solution of sulfuric acid and hydrogen peroxide for 10 min prior to being thoroughly rinsed in deionized water and dried with compressed dry air.

Oxygen plasma treatments were performed using a reactive ion etching tool (Nordson March CS-1701). The tool allows for precise experimental control of treatment power and exposure time. All experiments used the tool’s independent pressure control mode to regulate the oxygen gas pressure in the chamber to 500 mT during treatment.

### 2.2. Contact Angle Measurement

**Figure 1:**
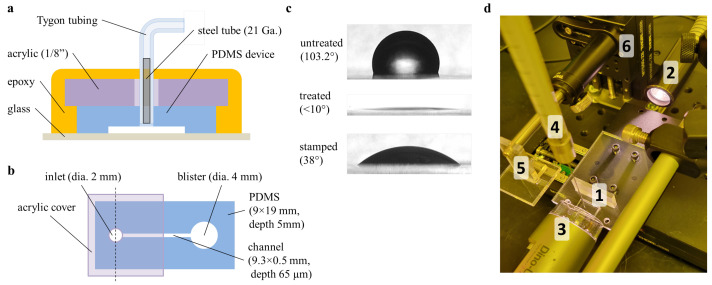
Bond strength estimation and measurement approaches. (**a**) A cross-section showing epoxy and acrylic scheme designed to provide structural support during high-pressure burst testing, (**b**) the footprint for blister burst testing devices, and (**c**) representative images of a 20 W, 3 s oxygen plasma treatment on the contact angle of PDMS and recovery after oligomer stamping. The contact angle measurement and stamping rig (**d**) positions a sample (1) between dispersed incandescent background lighting (2) and a digital microscope (3). Liquid samples are dispensed from a mounted syringe (4). The automated translation stages (6) provide uniform and precise stamping (5).

Because the effectiveness of oxygen plasma surface treatment degrades over time [1], the contact angle measurement setup (Figure 1d) was located physically close to the plasma etching tool. Immediately following plasma treatment, 8 mm × 12 mm × 3 mm PDMS slabs were transferred to a fixture and secured for stamping or measurement. To ensure uniformity of stamping pressure and time, a programmable motorized translation stage (Thorlabs MTS50-Z8) was used to position and stamp the samples. Sessile drops of less than 2 µL of deionized water were deposited on the surface manually via a 23 gauge syringe (Trajan 000800). High resolution videos of the droplet placements were captured by a horizontally mounted digital microscope (Dino-Lite AF3113T) under approximately 20× magnification. Videos were trimmed to capture only the frames where the droplet was determined to be dynamically spreading, thus capturing only the advancing contact angle and more accurately reflecting the surface energy after treatment [13,14]. A subpixel edge detection algorithm was used to fit the droplet edge to a 2nd -order polynomial and subsequently extract left and right contact angles for each frame of the trimmed video [15]. Reported measurements reflect the average extracted angle over the valid frames of each droplet spreading. For untreated PDMS, the spreading contact angle for deionized water was found to be 108.3°.

### 2.3. Blister Testing

To directly measure PDMS–glass bond strength, a pressurized blister burst testing method was developed. PDMS devices (5 mm thickness) with simple closed channels were bonded to glass substrates. A girdle of acrylic plastic and epoxy (3M DP100) was constructed to secure the inlet tubing at pressures exceeding 100 psi (Figure 1a). The 4 mm-diameter termination port on each device was not reinforced, causing inflation of nitrogen gas to exert force on the blister edge, eventually leading to breakdown in bond integrity at high pressures. Figure 1b indicates the complete device specifications, since burst testing is known to be dependent on the blister geometry [16]. Video feeds from a digital microscope (Dino-Lite AF3113T) and a webcam directed at the pressure gauge were synchronized and analyzed to detect the critical pressure at which bond breakdown first occurred. For elastomer stamping experiments, the mechanized rig used for contact angle measurements (Figure 1d) was used to stamp the blister portion of the sample, ensuring uniform stamping pressure and duration.

### 2.4. Valve Design

**Figure 2:**
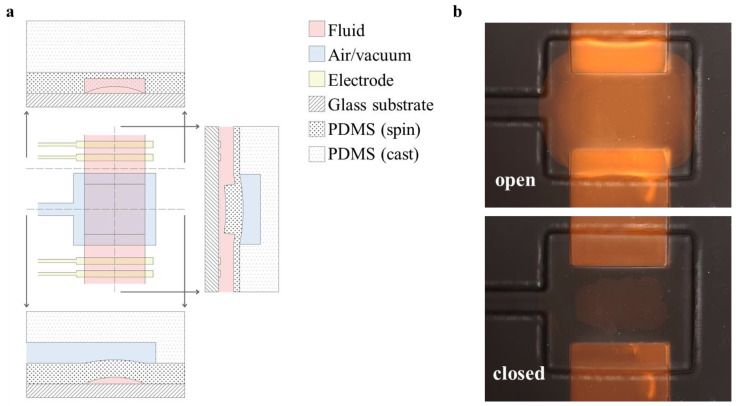
(**a**) Diagram of the valve construction showing three cross-sections of the valve operation in the open/vacuum states and (**b**) fluorescent microscopy images (10×, false color) of a valve in the open and closed positions with rhodamine dye flowing in the channel.

**Figure 3:**
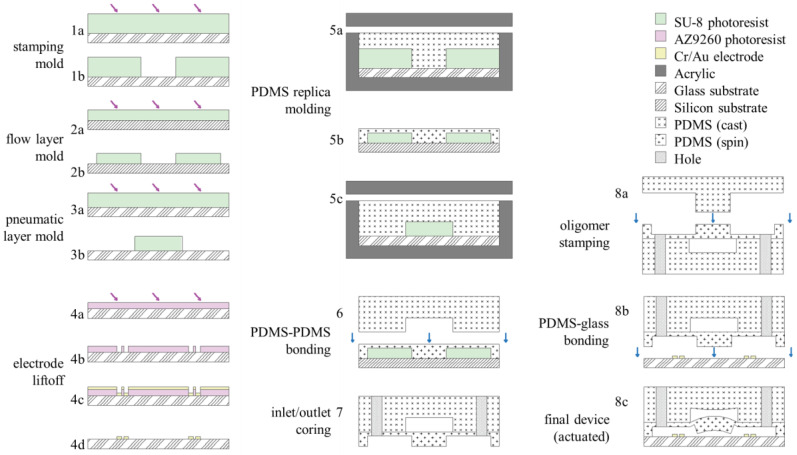
Device fabrication. The (**1a**–**b**) stamp mold, (**2a**–**b**) flow layer mold, and (**3a**–**b**) pneumatic layer mold are fabricated in standard photolithography using SU8. (**4a**–**d**) Electrodes are patterned on a glass substrate by liftoff photolithography and Cr/Au sputter deposition. (**5a**,**5c**) PDMS is cast-molded for the stamp and pneumatic layers and (**5b**) spincoated to form a thin membrane on the flow layer. (**6**) Flow and pneumatic layers are aligned and bonded by oxygen plasma treatment. (**7**–**8c**) The final device is plasma treated, stamped, and aligned and bonded over the electrodes.

Negative-pressure elastomeric valves with active areas of 400 × 500 µm (Figure 2b) were fabricated using a PDMS molding process and standard photolithographic techniques. SU8 was deposited to a depth of 65 µm for both the flow and pneumatic-control layer master molds (Figure 3 (2a–3b)). The valve-stamping mold was fabricated with a feature depth of roughly 100 µm to accommodate leveling errors in alignment (Figure 3 (1a,b)). The pneumatic layer was cast-molded in an acrylic/glass fixture (Figure 3 (5c)), which ensured a uniform thickness of 5 mm and sufficient flatness of the top surface for subsequent alignment steps. A relatively thick device depth of 5 mm was found to be favorable for handling the pneumatic layer with minimal stretching or warping. Special care was taken to avoid deforming the treated PDMS to avoid mechanical stress, which can accelerate recovery of the surface treatment [1] and improve alignment.

**Figure 4:**
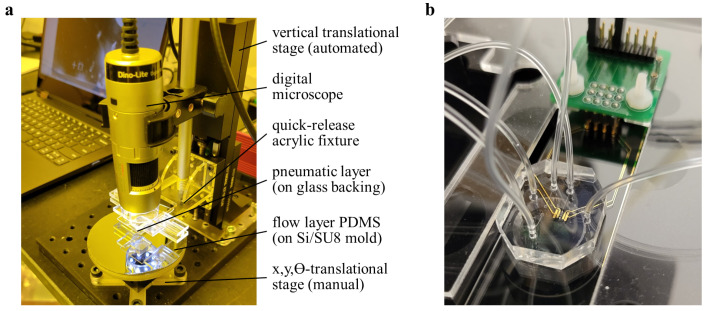
(**a**) Alignment rig for PDMS–PDMS bonding, oligomer stamping, and PDMS–glass bonding. (**b**) Completed microfluidic device featuring three normally closed valves mounted on an inverted microscope and electrically contacted.

The flow layer mold was spincoated with uncross-linked PDMS at 2000 rpm for 6 s, forming thin (approximately 40 µm) membranes at the valve sites (Figure 3 (5b)). After curing, the cast pneumatic layer was released from the mold, while the flow layer remained on the wafer. Both layers were oxygen plasma treated simultaneously at 20 W for 30 s. A glass backing was applied to the pneumatic layer PDMS before the layers were quickly (less than 5 min) aligned and brought into light contact (Figure 3 (6)). The alignment rig (Figure 4a) was designed to quickly release the glass backing so that the device could be transferred to a hotplate, completing the bonding with a 10 min bake at 80 °C. Once the bond was completed, the device was released from the wafer mold, trimmed to size, and cored with a 1.5 mm biopsy punch to create inlet ports (Figure 3 (7)).

Finally, the devices were treated, stamped, aligned, and bonded to glass slides patterned with gold electrodes (Figure 3 (8a–c)). The untreated elastomeric stamp was mounted on a glass backing and pre-aligned with the device to be bonded in order to reduce the total plasma treatment recovery time. The PDMS device and glass substrate were simultaneously treated with oxygen plasma at 20 W for 30 s. Treated PDMS was brought into alignment with the valve stamp and contacted 3 times for 3 s using the automated mechanized stage. The device itself was then attached to a glass backing, aligned to fiducial markings on the glass substrate, and baked on a hotplate at 80 °C for more than 10 min. After cooling to room temperature, vacuum was pulled to release stamped areas, completing the valve fabrication.

The electrodes were fabricated on glass microscope slide substrates using a standard liftoff process (Figure 3 (4a–d)). The electrodes were patterned lithographically using AZ9260 photoresist (AZ Electronic Materials) at a thickness of 6.5 µm. An adhesion layer was formed by first sputtering chromium to a depth of 15 nm before further sputtering of gold electrodes to a depth of 210 nm. Liftoff was performed in heated Microposit Remover 1165 (Dow Chemical) overnight.

### 2.5. Valve Characterization

Four-point electrical impedance measurement was performed using a Keithley 6221 (Tektronix) alternating current source in conjunction with a digital lock-in amplifier (Stanford Research Systems SR830). The injected current amplitude was adjusted to match the dynamic range of the amplifier in each experiment and ranged from 10 nA to 1 µA. Complex impedance was recorded over a frequency spectra from 100 Hz to 10 kHz. Source frequency was swept in 30 logscaled values over a total of approximately 180 s, allowing settling time inversely proportional to source frequency, and 10 data points were averaged per frequency set point. Both instruments were controlled over serial interface and programmed via LabVIEW software 2022 Q2 (National Instruments, Austin, TX, USA). A four-point measurement scheme serves to negate the impact of lead and electrode contact resistances on the impedance measurement. Figure 4b shows the electrical interface to a microfluidic test device. Contact pads on the devices were probed using spring-loaded contact pins (Millimax Series 825), which were routed to BNC connections through a printed circuit board. All electrical characterization of valve performance was conducted using Dulbecco’s phosphate buffered saline solution (DPBS, Thermo Fischer Scientific, Waltham, MA, USA) as the only medium.

Dynamic pressure control for valve characterization was provided by an OB1 Mk3 regulator (Elveflow) with pressure stability and resolution below 0.1 mbar. This multichannel regulator was used to drive both fluid flow and positive pressure pneumatic actuation of the elastomeric valves. Vacuum pressure was generated by a portable 75 Torr vacuum pump (N810FTP, Ideal Vacuum Products, Albuquerque, NM, USA). The flow of gas (biological atmosphere mixture, Airgas, Anchorage, AK, USA) was directed by solenoid valves controlled by a MUXW manifold (Elveflow, Paris, France). Coordination of all pressure control systems was programmed in LabVIEW.

Microscopy images of the final normally closed valves were captured using an inverted fluorescence microscope (EVOS FL Auto 2) in phase contrast mode.

## 3. Results and Discussion

### 3.1. Optimal Bonding Conditions

Contact angle measurements serve as a useful proxy for eventual bond strength. Plasma treatment of the surface renders it hydrophilic, so a lower deionized water contact angle correlates with more-effective surface treatment. Likewise, the contact angle is inversely proportional to bond strength. However, for stamped samples, an increased contact angle is ideal as it indicates reduced bonding in the valve area.

The effects of oxygen plasma treatment for bonding on non-stamped PDMS have been previously investigated [2]. It was shown that the contact angle reaches a minimum near a plasma treatment power of 20 W over a total exposure time of 30 s. However, the accuracy of telescopic contact angle goniometers in general decreases significantly at lower contact angles [14], and our setup was unable to detect any statistically significant difference in angles resulting from the reasonable range of treatment conditions on unstamped samples. Droplets deposited on treated surfaces exhibited strong wetting with near-zero contact angles. These results were significantly lower than those of the previous study [2], though others have also assessed the contact angle under plasma treatment to be too near zero to report any other result [12].

**Figure 5:**
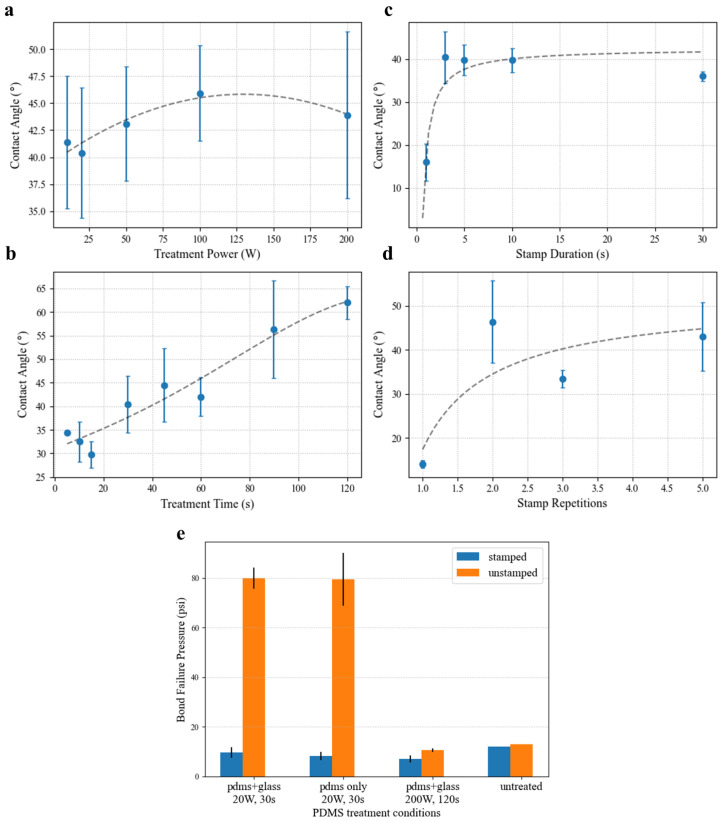
(**a**–**d**) Spreading contact angle measurement results for deionized water on PDMS samples treated by oxygen plasma and subsequently stamped with untreated PDMS. (**a**) Plasma treatment power was investigated at a fixed treatment time of 30 s and 3 s stamp duration, and (**b**) treatment duration was swept at 20 W treatment power and 3 s stamp duration. Similarly, experiments investigating the (**c**) stamping duration and (**d**) number of stamping repetitions used treatment conditions of 20 W for 30 s exposure. (**e**) Additionally, blister bursting tests were conducted to evaluate the pressure at which bonded devices failed.

Figure 5 shows the results of oligomer stamping on the PDMS bond strength via treatment with oxygen plasma. The effectiveness of the stamping approach is clearly demonstrated, with the contact angle recovering nearly 40° for most treatment conditions. As shown in Figure 5a, for 30 s treatments at 500 mT, the measured contact angle shows little dependence on the treatment power. While the average values generally show constant contact angle recovery compared to the previously published unstamped results (i.e., a similar shape with a minimum near 20 W), the standard deviations of the measurements (N ≥ 3 drops, 6 angles) are quite large. Though attempts have been made to control as many variables in the stamping process as possible (i.e., duration, pressure, angle) through automation, the effect on the surface treatment still seems to be quite variable. One difference that was observed between samples that is particularly difficult control is the manner in which the stamp and surface under test separate. For the large surfaces necessary for droplet spreading, the PDMS samples tended to peel apart in a wavefront that originated at inconsistent locations throughout these experiments. As a result, some areas of the samples may have experienced more mechanical perturbance, as the final region of contact often sprang apart elastically.

Contact angle results examining the effect of plasma treatment time with source power of 20 W (Figure 5b) showed a much stronger dependency. Again, the minimum contact angle matches the previously reported minimum for unstamped PDMS near 30 s. This minimum is expected for unstamped PDMS, as insufficient exposure produces fewer of the silanol chains needed for bonding, and increased overexposure results in the formation of a brittle silica-like layer [17,18]. Oligomer stamping likely acts by removing or reorienting the activated surface groups and should not be able to reverse the formation of the silica layer. Rather, its action seems to be proportional to the concentration of activated surface groups, resulting in similar contact angle curve shapes.

The stamp contact duration was shown to play a significant part in the effectiveness of the oligomer stamping approach in selectively removing the PDMS surface treatment. Figure 5c shows that under test conditions of 20 W and treatment time of 30 s, the optimal stamping time should exceed 1 s, but contact lasting longer than 3 s offers no further benefit. While the mechanism of oligomer stamping does not seem to occur instantaneously, contact angle testing in Figure 5d shows that the action can be repeated. Making stamp contact with the treated surface multiple times further decreased contact angle in the contacted area. However, this approach showed diminishing returns after 3 repeated stamps. So long as alignment can be retained between successive contacts (requiring both the stamp and the device to be well-fixed on their backings), multistamping is an effective way to increase the bond strength margin between the treated and stamped areas without compromising spatial resolution.

Blister bursting tests assessing bonding strength (Figure 5e) confirm contact angle results, showing an 8× decrease in bond strength between treated and stamped samples for the optimal bonding conditions of 20 W and 30 s. Interestingly, treatment of the glass substrate seems to have little effect on the final bond strength. This investigation found that plasma-treating the substrate seems primarily to decrease the variability in the bonding process. This advantage could likely be attributed to removal of any possible surface contamination by plasma treatment rather than to surface activation. Burst testing further demonstrated that the conditions at which the stamped contact angles were greatest (high treatment power and long exposure) are unsuitable for device bonding. In fact, both stamped and unstamped samples exposed to 200 W oxygen plasma for 2 min exhibited poorer substrate adhesion than untreated PDMS.

Oligomer stamping is clearly an effective means of spatial patterning of plasma surface treatment on PDMS. For optimal recovery of non-bonding areas, stamps should contact the surface for more than one second, and contact should be repeated at least three times. Following these recommendations, sufficient removal of the surface treatment to prevent bonding can be achieved for any treatment conditions. Thus, for normally closed PDMS valves, the surfaces should be treated to optimize the performance of the bonding surfaces rather than to ensure valve release.

### 3.2. Valve Performance

**Figure 6:**
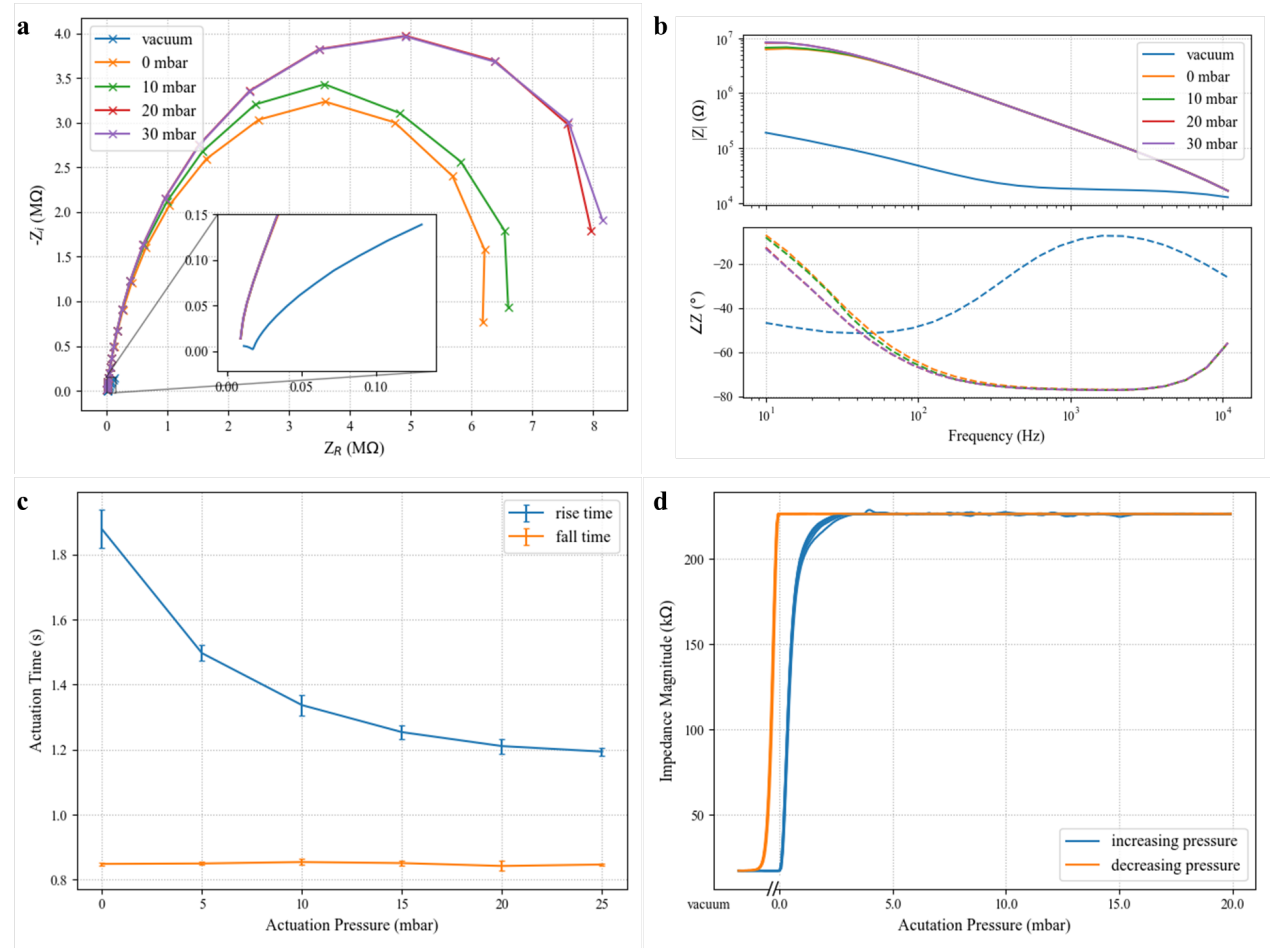
Impedance spectra for a valve in DPBS at various actuation pressures in both (**a**) Nyquist (**b**) and Bode plots. The current source (1 µA for vacuum, 10 nA for all other sweeps) was swept through evenly logspaced frequency points from 10 Hz to 10 kHz. (**c**) Valve actuation time was measured between 10% and 90% of the settled values of complex impedance magnitude after 60 s. A 1 kHz, 100 nA current stimulus was used to probe valve impedance. (**d**) Valve hysteresis was observed by step-wise increases to the actuation pressure from 0 to 20 mbar then ramping the pressure back to 0 mbar before returning to vacuum. The corresponding impedance through the valve was recorded for a 100 nA signal at 1 kHz sampled continuously every 250 ms. Five complete cycles are overlapped in the plot.

Fabrication on glass is ideally suited to integrate planar microelectrodes into valved microfluidic devices. In the presence of a highly conductive medium, such as DPBS, the electrical impedance seen across the valve site serves to characterize the effectiveness of the valve sealing [19], and the impedance ratio between the open and closed states of the valve signifies the electrical isolation it provides at each frequency. Figure 6a,b show the characteristic complex impedance spectra for a representative valve at various actuation pressures. At low frequencies, the valve impedance magnitude exceeds 8 MΩ for positive actuation pressures greater than 10 mbar when the valve is sealed. In contrast, the measured impedance magnitude for the valve in the open state (vacuum) is only 190.0 kΩ at 10 Hz, a 35-fold difference. At 30 mbar actuation pressure, the valve in the closed state exhibits a maximum of 55.6-times greater impedance magnitude over the open state, showing that such normally open valves are suitable to provide reversible electrical isolation of different microfluidic compartments.

In the closed state, the valve impedance curves are bandwidth-limited by the cabling (BNC) connecting benchtop instruments to the device under test. The closed valve impedance closely matches a single-pole model with a cutoff frequency of 31 Hz. At 30 mbar, it can be modeled by a resistor and capacitor in parallel with equivalent values of 8.2 MΩ and 0.62 nF, respectively. Resistive load tests have verified that this parasitic capacitance is introduced in the cabling rather than the device or interconnect. The valve barrier then represents a constant resistance over the biologically relevant spectrum. Even in its resting state (0 mbar), the valve represents an extracted resistance of 6.4 MΩ, making these devices useful for applications characterizing analytes across a broad frequency range (e.g., dielectric spectroscopy flow cytometry) or those using high-frequency stimulation.

Ionic conduction in the open valve state also experiences significant degradation across the measured frequency range. The vacuum curve shape in Figure 6a is reminiscent of the characteristics of a series connection of two parallel resistor–capacitor pairs. This model matches previously studied electrical models for the electrode–fluid interface, where a large capacitance arises from the electric double layer formed by free ionic species at the interface, and a resistance models redox reactions responsible for charge transfer [20,21].

Valve actuation time is shown to be a function of the pneumatic actuating pressure in Figure 6c. The rise time corresponds to the impedance magnitude rise observed when positive pressure is applied to a valve’s pneumatic chamber and is calculated as the time difference between 10% and 90% of the total impedance rise after 60 s. As would be expected, driving the system at higher pressures causes more rapid deflection of the elastomer membrane, resulting in shorter rise times. However, diminishing returns in rise time are observed beyond 20 mbar, and valves should be operated at the minimum acceptable pressure level to preserve longevity. Fall times (the time to drop from 90% of the sealed impedance to 10% once vacuum is applied) do not exhibit dependency on the previous actuation pressure since all valves are well-sealed after 60 s, and an identical vacuum pressure is applied in all cases. Presumably, decreased vacuum strength would result in increased falling valve actuation times, but this response was not investigated. Figure 6b shows the valve hysteresis for a 1 kHz signal as a function of the actuation pressure. We posit that this hysteresis effect is primarily mechanical in nature. When vacuum is released on the valve, the elastic membrane begins sealing immediately, achieving a 10-fold impedance increase with less than 1 mbar of applied pressure. The valve impedance plateaus at less than 5 mbar of positive pressure, indicating that the membrane has completely sealed against the substrate. Once sealed, the elastomer is in a low-energy state, and electrical isolation is retained upon decreasing the actuation pressure back to zero. For low-power solutions that rely on solenoid valves to apply pressure, this result implies that a scheme whereby the actuation pressure is briefly pulsed instead of held continuously is feasible.

**Figure 7:**
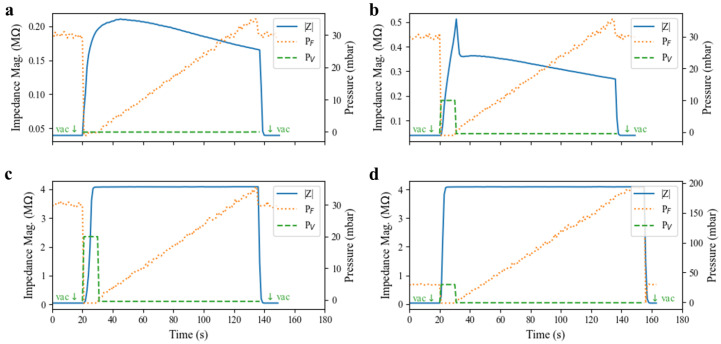
Valve resilience to fluid flow pressure (P${}_{F}$) in the channel characterized by electrical impedance through the valve at 100 Hz. Valves were initially in an open state before the pneumatic valve pressure (P${}_{V}$) was pulsed at (**a**) 0 mbar, (**b**) 10 mbar, (**c**) 20 mbar, and (**d**) 30 mbar for 10 s. As the back-pressure on the valve was then ramped linearly, leakage current through the valve could be observed as a decay in valve impedance magnitude. Experiments began and ended with P${}_{V}$ pulled to vacuum (approximately −100 mbar, denoted by downward arrows).

The valve dynamics are explored in Figure 7, and such a pulsed actuation scheme is demonstrated. The ability of the valve to create and maintain a complete seal in the presence of positive pressure applied to the fluid channel was tested. A test valve was first closed for 10 s using varying actuation pressures (0, 10, 20, and 30 mbar) before the pneumatic pressure on the valve was released and positive pressure on the fluid channel flowing into the valve was increased linearly every 3 s. The electrical impedance was measured across the valve to monitor the seal quality and any leakage current. In Figure 7a, when zero positive pressure was applied to the valve, its elastomeric membrane slowly returned to its resting state against the substrate, resulting in a gradual increase in impedance. However, the force generated by the elastomer relaxation was likely insufficient to expel all of the conductive fluid under the valve area, resulting in a poor seal and a small fluid leakage current under the valve. The leakage current expanded at higher driving pressures, resulting in declining electrical impedance. Valve impedance decreased to 90 percent of its maximum value at less than 24 mbar, suggesting that for operation without positive pressures, some amount of fluid leakage must be acceptable (e.g., a peristaltic pump or control of flowing cell suspensions) or the device must operate at very low pressure. At 10 mbar actuation pressure (Figure 7b), the valve impedance rose quickly, but it was still insufficient to form a complete seal in the 10 s actuation window. A small leakage current was still present, as evidenced by the similarity of the post-actuation curve shape with that of the 0 mbar case. Figure 7b indicates a trade-off between the actuation pressure and actuation window in order to form complete seals in this pulsed control scheme. For a 10 s actuation window, 20 mbar actuation pressure is sufficient to fully seal the channel, as evidenced by Figure 7c. Further, Figure 7d demonstrates that for a 10 s pulse of pressure at 30 mbar, the valve forms a sufficient seal to the substrate to withstand a 200 mbar flow pressure (the full range of the experimental pressure controller) without any leakage current forming. Presumably, these valves can withstand even greater fluid pressure given sufficient actuation pressure and time to form a tight seal to the substrate.

### 3.3. Limitations and Future Work

Traditional lithographic alignment processes are not optimized for “soft” materials. PDMS alignment for bonding in particular presents a variety of challenges, namely (1) the possibility of layers stretching or drooping, (2) challenges related to leveling soft surfaces, and (3) more-pronounced thermal contraction.

The elastic nature of PDMS permits stretching, warping, and drooping of thin layers if not rigidly backed during alignment. Ideally, layers should be cured against transparent backing and not released until alignment is complete. Care must be taken, however, to ensure that the backing itself is either sufficiently light or well-balanced enough that it does not exert significant levering force on the device after alignment while the bond is developing.

Contrary to normal photoresist alignment processes, where cross-linked photoresist may be contacted to the mask in the stage-leveling step prior to alignment, the treated PDMS surface must not contact its bond target prior to final contact. Further, the compressibility of PDMS makes leveling procedurally difficult, as a freely rotating stage may compress the device rather than adjusting its angle of alignment.

If the challenges of (1) and (2) are addressed and good alignment is achieved, further issues can arise due to the trapping of air. When both surfaces are leveled, contact can occur at multiple points across the device, causing air pockets to arise in the bonding interface. The gas permeability of PDMS can help evacuate some of these air pockets during the heated curing phase, but they often result in areas of decreased bond strength.

Many of these challenges could be addressed through the continued development of a customized alignment and plasma treatment rig. An ideal tool might operate similarly to a mask aligner, where the UV lamp is replaced by a plasma source. The ability to align soft layers and fix their position during plasma treatment would alleviate many of the noted limitations of this approach. Practically, alignment challenges, rather than bond strength, can lead to low yield of valved devices. In theory, spatial resolution of the approach is limited only by lithographic resolution and alignment quality.

### 3.4. Conclusions

A spatially selective bonding method has been demonstrated to fabricate normally closed microfluidic valves on glass substrates. This oligomer stamping technique had not previously been demonstrated on glass and prevents the need to actuate valves during the bonding process, thereby increasing fabrication reliability and enabling large-scale integration. The oligomer stamping process itself has been examined in detail using contact angle measurements to predict bonding strength. The stamping mechanism was found to produce a roughly constant recovery of the PDMS surface hydrophobicity over a wide range of exposure parameters. Further, the oligomer interaction with activated surface groups does not occur instantaneously; rather, stamping to remove plasma surface treatment should maintain contact for greater than 3 s and may be repeated to ensure maximum recovery of hydrophobicity. Blister burst testing showed that, in practice, this stamping technique is sufficient to prevent bonding under any surface treatment conditions, so oxygen plasma treatment should be optimized for efficient bonding of the device area rather than minimum bonding in the valve area, i.e., 20 W exposure for 30 s.

The elastomeric valves fabricated using this optimization require only two PDMS layers (and no additional chemicals or fabrication tools) and permit convenient integration of electrodes into a microfluidic device. Integrated electrodes enable monitoring of valve behavior among a number of other useful applications. Here, electrical impedance measurements through the valves have demonstrated reversible electrical isolation beyond 8 MΩ at exceptionally low actuation pressures (less than 30 mbar). Further, a pulsed actuation scheme was explored, in which positive pressure is applied briefly to seal the valve against the substrate before releasing pneumatic pressure to conserve operational energy. Electrical measurement verified that valves pulsed in this manner at 30 mbar for 10 s were able to withstand channel back-pressures greater than 200 mbar without leaking. The presented microcontact method of valve fabrication is suitable for dense integration of many normally closed elastomeric valves on glass substrates, enabling applications where optical imaging or electrode integration are critical.

## Data Availability

All data presented will be made available through reasonable request of the corresponding author.

## Abbreviations

The following abbreviations are used in this manuscript:

PDMS	polydimethylsiloxane
DPBS	Dulbecco’s phosphate buffered saline
BNC	Bayonet Neill–Concelman
